# Small molecule metabolite biomarkers for hepatocellular carcinoma with bile duct tumor thrombus diagnosis

**DOI:** 10.1038/s41598-018-21595-4

**Published:** 2018-02-19

**Authors:** Weifeng Tan, Jingquan He, Junliang Deng, Xinwei Yang, Longjiu Cui, Rongzheng Ran, Guangwei Du, Xiaoqing Jiang

**Affiliations:** 1 Department of Biliary surgery, Eastern Hepatobiliary Surgery Hospital, The Second Military Medical University, Shanghai, 200438 China; 2 Department of Integrative Biology and Pharmacology, University of Texas Health Science Center at Houston, Houston, TX 77030 USA; 3 Department of Chemistry, Fudan University, Shanghai, 200433 China; 4 Department of Surgery, University of Pittsburgh School of Medicine, Pittsburgh, PA 15213 USA

## Abstract

Hepatocellular carcinoma with bile duct tumor thrombus (BDTT) is a malignant disease. The most commonly used diagnosis methods for BDTT are MRCP/ERCP, ultrasonic diagnosis or CT scan. However, BDTT is often misdiagnosed as other bile duct diseases, such as extrahepatic cholangiocarcinoma (EHCC), choledochal cyst (Cyst) and common bile duct stone (Stone). Diagnostic methods, which are more accurate and less destructive, are urgently needed. In this paper, we analyzed the small molecule metabolites in the serum of BDTT, Stone, Cyst and EHCC patients and normal people using untargeted GC-MS, and identified 21 metabolites that show different levels among different samples. Using targeted UHPLC-QQQ-MS analysis, we found that several metabolites are significantly changed. ROC curve analysis revealed two metabolites, L-citrulline and D-aspartic acid, as potential biomarkers that can distinguish BDTT from other bile duct diseases.

## Introduction

Hepatocellular carcinoma (HCC) is one of the most aggressive cancers worldwide^1^. Though the vascular invasion has been well described in some HCC patients, the occurrence of bile duct tumor thrombus (BDTT) in those patients is still poorly understood^2–4^. HCC with BDTT is caused by the migration of hepatocarcinoma cancer cells from liver to bile duct that subsequently induces the obstruction of bile duct. HCC with BDTT is a very malignant disease with poor treatment^5^. HCC patients with BDTT have lower overall survival rate than HCC patients without BDTT^6^. In 2014, Ashwin Rammohan and colleagues reported that there’s 39 HCC patients developed BDTT in 426 patients with HCC^7^. Although the occurrence of BDTT is not too high, the rate of misdiagnosis of HCC with BDTT is as high as 16%^8^. The most important reason for misdiagnosis of BDTT is its similarity to other bile duct diseases, such as extrahepatic cholangiocarcinoma (EHCC), choledochal cyst (Cyst) and common bile duct stone (Stone), due to similar clinical appearance and imaging properties^8^.

Surgical treatment for HCC is considered as the most efficient method^2^, including those with BDTT^9^. Recently, there’s also a paper reported using radiofrequency (RF) ablation as an alternative therapeutic method^10^. However, misdiagnosis of BDTT may lead to inappropriate treatments, resulting in poor survival of those patients. The current diagnosis methods for BDTT are Magnetic resonance cholangiography (MRCP)/ Endoscopic Retrograde Cholangiopancreatography (ERCP), ultrasonic diagnosis, computerized tomography (CT) scan^11^. These medical imaging methods have decreased the rate of misdiagnosis of BDTT. However, they are still not so efficient for BDTT diagnosis. To solve this problem, some investigators are focusing on searching for new markers for diagnosis, such as some stem cell markers^12^. However, a faster, more accurate and less destructive diagnosis method is still highly demanded.

The pathological conditions resulted from human diseases change metabolic activity of many tissues that often can be detected by the alteration of changes of small metabolites in the blood. Current technical progresses in metabolomics have allowed systematical analyses of a large number of metabolites in multiple samples, and is frequently used in quantitatively investigating metabolites and their dynamic changes in response to extracellular stimulation or in disease progression^13^. In this paper, we applied Gas Chromatography-Mass Spectrometry (GC-MS) and Mass Spectrometer coupled to high-pressure chromatography (UHPLC-QQQ-MS) methods to analyze the small molecule metabolites from the serum samples of EHCC, Cyst, Stone and BDTT patients. We found that several metabolites are significantly changed in BDTT patients compared with other groups. Furthermore, ROC curve analysis showed that the combination of L-citrulline and D-aspartic acid shows a very high sensitivity and specificity in BDTT patients. Thus they are potential biomarkers for BDTT diagnosis that can distinguish BDTT from other three diseases.

## Materials and Methods

### Patient information

The serum samples for the initial GC-MS analysis were obtained from normal individuals and patients with different types of disease (Supplementary Table 1), including 20 bile duct stone patients, 23 choledochal cyst patients, 30 extrahepatic cholangiocarcinoma patients, 7 HCC with BDTT patients, and 22 normal individuals. Some newly collected patient serum samples combined with some old patient samples which are not stored for very long time are used for the secondary UHPLC-QQQ-MS analysis (Supplementary Table 2) to confirm the GC-MS data. All of these patient samples were obtained between January 2009 and December 2012 from Eastern Hepato-Biliary Surgery Hospital, Shanghai, China. Informed consents were signed by all participants, and this study has been approved by the Committee on Ethics of Biomedicine Research, Eastern Hepatobiliary Surgery Hospital, Second Military Medical University. All methods used in this paper were carried out in accordance with the official recommendations of the Chinese Community Guidelines.

### Small molecule metabolomics analysis

0.4 ml methanol and 50 μl of L-2-Chlorophenylalanine (0.1 mg/ml stock in dH2O, Shanghai Hengbai chemdrug biotechnology Co., Ltd, Shanghai, China), which is used as an internal standard, were added to 100 μl sample serum, and vortex for 10s. After Centrifugation for 10 min at 12000 rpm, 4 °C, 400 μl supernatant was transferred to a GC-MS glass vial, and dried in a vacuum concentrator. 80 μl methoxyamination reagent (20 mg/ml in pyridine) was then added to the vials and shake at 37 °C for two hours. The samples were then mixed gently with 0.1 ml BSTFA reagent (1% TMCS, v/v, REGIS Technologies.Inc. USA), and shaked for 1 h at 70 °C, before they were cooled down for GC-MS analysis.

GC-MS analysis was performed using an Agilent 7890A gas chromatograph system (Agilent, USA) coupled with a Pegasus HT time-of-flight mass spectrometer (LECO, USA). The system utilized a DB-5MS capillary column coated with 5% diphenyl cross-linked with 95% dimethylpolysiloxane (30 m × 250 μm inner diameter, 0.25 μm film thickness; J&W Scientific, Folsom, CA, USA). A 1 μL aliquot of the analyte was injected in splitless mode. Helium was used as the carrier gas, the front inlet purge flow was 3 ml/min, and the gas flow rate through the column was 20 ml/min. The initial temperature was kept at 50 °C for 1 min, then raised to 330 °C at a rate of 10 °C/min, then kept for 5 min at 330 °C.The injection, transfer line, and ion source temperatures were 280, 280, and 220 °C, respectively. The energy was −70 eV in electron impact mode. The mass spectrometry data were acquired in full-scan mode with the m/z range of 85–600 at a rate of 20 spectra per second after a solvent delay of 366s.

UHPLC-QQQ-MS analyses was performed to confirm metabolites with significant changes in GC-MS results. Metabolites were extracted in methanol: acetonitrile (2:5) solution. For each metabolite, a standard curve was made by using a concentration gradient of 0.01–1 µg/ml. All the standards were purchased from Sigma-Aldrich (St. Louis, MO, USA). For targeted quantification, 5 µl samples were loaded on ACQUITY UPLC BEH HILIC VanGuard Pre-column (Waters, MA, USA) or ACQUITY C18 column (Waters).

### Data analysis

Chroma TOF4.3X software of LECO Corporation and LECO-Fiehn Rtx5 database were used for raw peaks extracting, the data baselines filtering and calibration of the baseline, peak alignment, deconvolution analysis, peak identification and integration of the peak area^14^. The RI (retention time index) method was used in the peak identification, and the RI tolerance was 5000. Metabolite spectra were normalized with the spectrum of internal standard. Then the standardized data was used for principal component analysis (PCA) and orthogonal partial least squares discriminant analysis (OPLS-DA). Kruskal-Wallis one-way ANOVA afollowed by Dunn’s or Holm-Sidak test was used to compare three or more groups. VIP > 1 (Variable importance in projection) was applied, and P < 0.05 was considered as significantly different. Then, Receiver Operating Characteristic (ROC) curve analysis was applied to determine the sensitivity and specificity of the differentially expressed metabolites for BDTT diagnosis.

## Results

### Patient characteristics

The serum samples from all the patients were analyzed, and the level of some molecules that are always used as markers for diagnosis are listed in Tables 1 and 2. The patients without tumors (Cyst and Stone) had normal level of alpha-fetoprotein (AFP), Carcinoembryonic antigen (CEA) and carbohydrate antigen 19–9 (CA19-9), which is used as markers for tumor diagnosis. EHCC and BDTT patients have increased CA19-9. Compared with EHCC, BDTT patients also showed increased AFP level. Several other factors, such as alanine transaminase (ALT), aspartate transaminase (AST), gamma-glutamyl transferase (GGT) and Alkaline phosphatase (ALP) are also increased in those patient serums, demonstrating obvious impairment of liver function. Though the increase of these serum markers is higher in EHCC and BDTT patients, it is still very difficult to distinguish among BDTT and other patients. Thus, to find new small molecule metabolites suitable for BDTT diagnosis is highly demanded.

**Table 1:** The information of patients of GC-MS experiment

	Gender No.(M/F)	Age	AFP (ng/mL)	CEA (ng/mL)	CA19-9 (U/mL)	TBIL (nmol/mL)	DBIL* (nmol/mL)	TBA**(nmol/mL)	TP^#^ (g/L)	ALB^##^ (g/L)	ALT (U/L)	AST (U/L)	GGT (U/L)	ALP (U/L)
Normal	12/10	38.3 ± 2.0	2.1 ± 0.3	1.7 ± 0.2	7.6 ± 1.0	9.4 ± 0.7	3.3 ± 0.3	5.0 ± 0.5	75.8 ± 1.2	42.6 ± 0.7	22.4 ± 2.3	19.0 ± 1.7	29.6 ± 2.7	76.3 ± 4.3
Stone	8/12	53.5 ± 3.4	3.1 ± 0.6	1.9 ± 0.3	70.9 ± 49.2	22.6 ± 6.3	14.6 ± 5.5	22.5 ± 9.6	71.5 ± 1.6	42.1 ± 0.9	67.2 ± 34.6	69.9 ± 38.6	260.6 ± 81.5	196.3 ± 45.7
Cyst	5/18	48.2 ± 3.1	3.2 ± 0.6	1.7 ± 0.2	26.2 ± 10.2	24.7 ± 9.0	15.6 ± 7.7	17.6 ± 9.1	70.0 ± 1.2	42.5 ± 0.8	30.1 ± 8.8	37.9 ± 15.9	119.3 ± 47.1	105.1 ± 27.5
EHCC	19/11	56.1 ± 1.8	2.9 ± 0.2	3.9 ± 0.9	178.7 ± 44.7	127.3 ± 22.3	100.6 ± 17.7	64.0 ± 10.7	66.8 ± 1.2	39.8 ± 0.8	146.9 ± 25.3	100.9 ± 16.4	639.2 ± 92.9	385.1 ± 70.7
BDTT	6/1	47.0 ± 4.4	7088.6 ± 6777.8	2.5 ± 0.8	278.7 ± 134.6	110.9 ± 42.5	86.4 ± 34.0	57.6 ± 21.7	68.5 ± 1.5	40.2 ± 1.3	310.9 ± 139.8	171.0 ± 56.5	730.9 ± 164.0	308.1 ± 82.9

*DBIL, direct bilitubin; **TBA, total bile acid; ^#^TP, tatal protein; ^##^ALB, albumin.

**Table 2:** The information of patients of UHPLC-QQQ-MS experiment.

	Gender No.(M/F)	Age	AFP (ng/mL)	CEA (ng/mL)	CA19-9 (U/mL)	TBIL (nmol/mL)	DBIL (nmol/mL)	TBA (nmol/mL)	TP (g/L)	ALB (g/L)	ALT (U/L)	AST (U/L)	GGT (U/L)	ALP (U/L)
Normal	5/5	37.2 ± 2.5	1.6 ± 0.3	1.4 ± 0.3	6.9 ± 1.5	7.6 ± 0.7	2.8 ± 0.3	5.2 ± 0.8	76.8 ± 1.7	43.1 ± 0.9	24.4 ± 3.0	20.3 ± 2.6	25.0 ± 3.0	70.5 ± 5.6
Stone	4/6	51.2 ± 5.7	2.6 ± 0.4	1.8 ± 0.4	14.9 ± 3.6	16.1 ± 3.5	8.4 ± 3.3	14.4 ± 8.8	69.9 ± 2.2	41.8 ± 1.5	40.1 ± 14.8	36.2 ± 12.7	212 ± 112.6	195 ± 78.6
Cyst	2/8	47.8 ± 5.6	2.2 ± 0.4	1.7 ± 0.3	43.4 ± 22.4	21.4 ± 7.9	12.5 ± 7.5	22.2 ± 17.6	71.3 ± 2.0	42.9 ± 1.3	45.4 ± 19.0	60.1 ± 36.1	136.8 ± 99.7	130.3 ± 61.1
EHCC	20/11	56.4 ± 1.7	2.8 ± 0.2	3.8 ± 0.8	177.5 ± 43.3	124.1 ± 21.8	98.0 ± 17.3	65.0 ± 10.4	66.6 ± 1.2	39.7 ± 0.8	148.4 ± 24.5	101.3 ± 15.9	636.5 ± 89.9	388.5 ± 68.5
BDTT	32/5	49.0 ± 1.6	1696 ± 1300	2.8 ± 0.3	241.2 ± 53.7	78.5 ± 15.5	57.4 ± 12.1	56.9 ± 14.5	71.5 ± 0.8	41.7 ± 0.6	152.5 ± 33.3	92.8 ± 15.3	515.6 ± 65.7	229.6 ± 25.3

### Identification of metabolite specifically altered in HCC with BDTT

**Figure 1:**
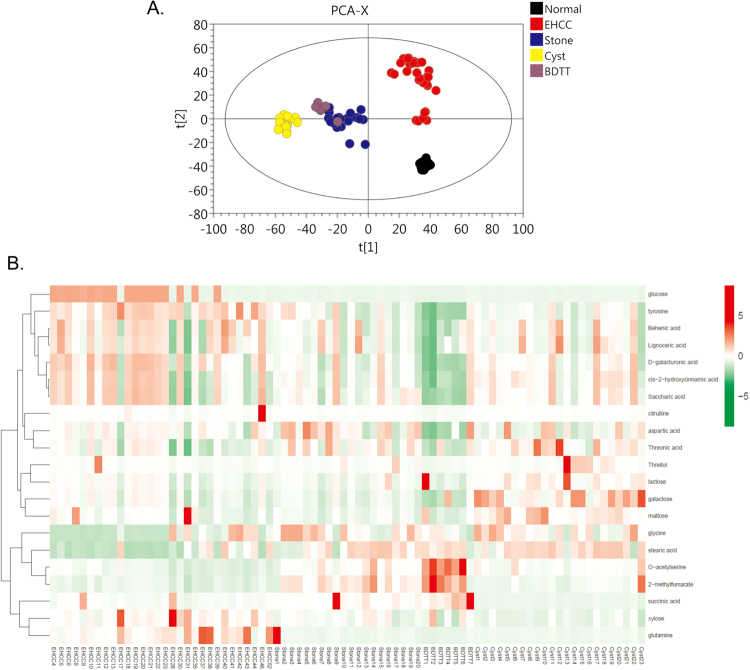
Untargeted GC-MS analysis identifies differentially changed metabolites in serum. (**A**) PCA score plot shows very well separation of metabolites profile among BDTT, EHCC, Cyst, Stone and control group. (**B**) The differentially expressed metabolites in GC-MS samples, and the relative level of metabolites was plotted in heat map. Red color represents increase of the metabolite, and green color means decrease of the level of metabolite.

By determining the small molecule metabolites, we totally detected 801 peaks in all of the patient serum samples in GC-MS analysis. After filtered by interquantile range denoising method, 623 peaks were confirmed to represent different small molecule metabolites (Supplementary Table 3). After normalization with the internal standard, the three-dimensional data were fed to SIMCA14 software package (Umetrics, Umea, Sweden). Based on PCA analysis and OPLS-DA analysis, the small molecule metabolite profiles are clearly separated in different groups (Fig. 1A), demonstrating that each disease has its unique metabolic profiles. Although BDTT and Stone group share some common metabolites, they can still be clearly separated by some metabolites (Fig. 1B).

### Confirmation of specifically altered metabolites in HCC with BDTT by UHPLC-QQQ-MS

**Figure 2:**
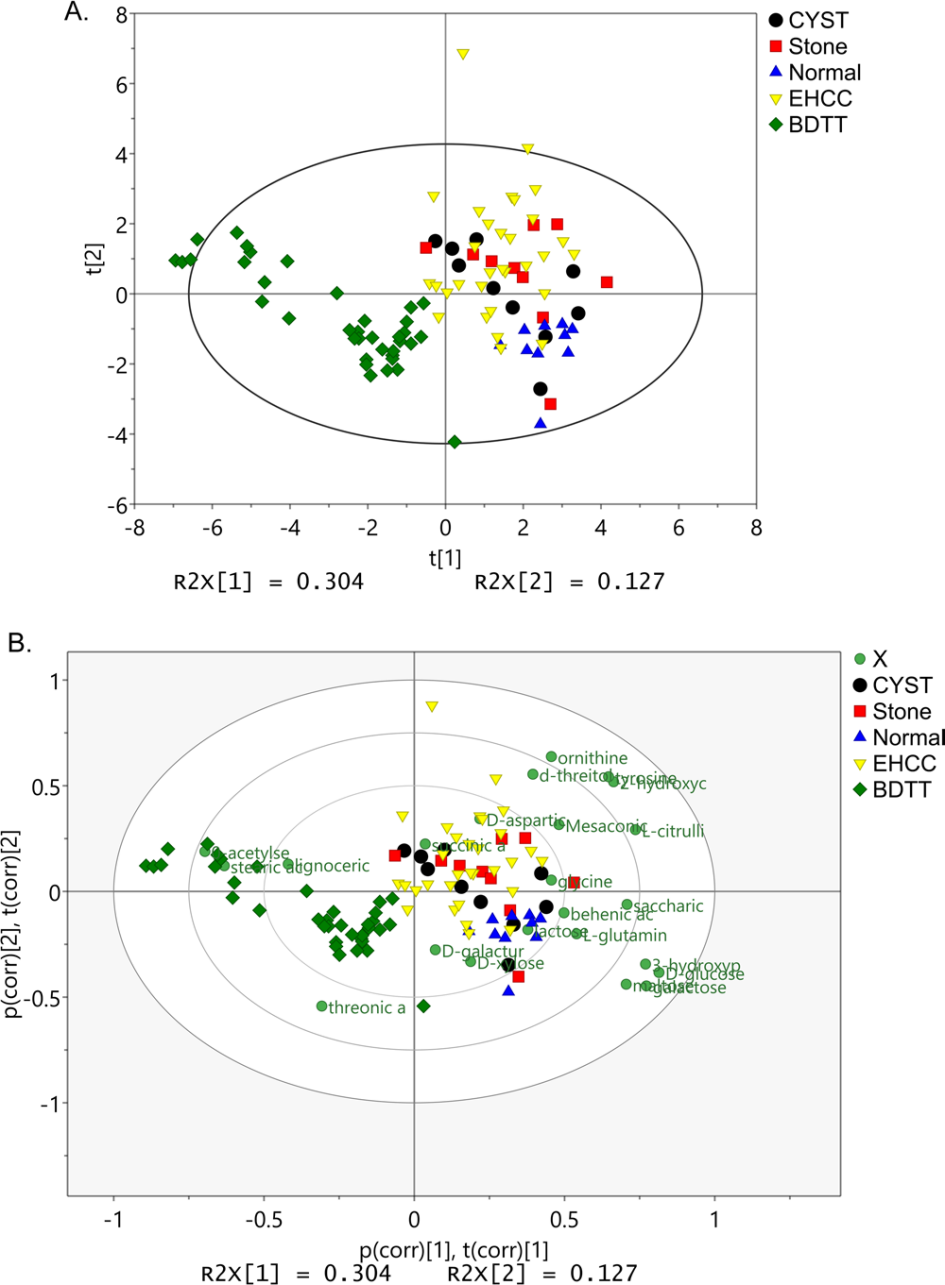
Targeted UHPLC-QQQ-MS analysis to confirm the level of metabolites. (**A**) Score plot of PCA model shows distinct distribution of BDTT group and other groups. The ellipse denotes the 95% significance limit of the model, as defined by Hotelling’s t-test. (**B**) Bi-plot analysis of concentration of the metabolites. Several metabolites were significantly decreased in BDTT group compared with other groups.

**Figure 3:**
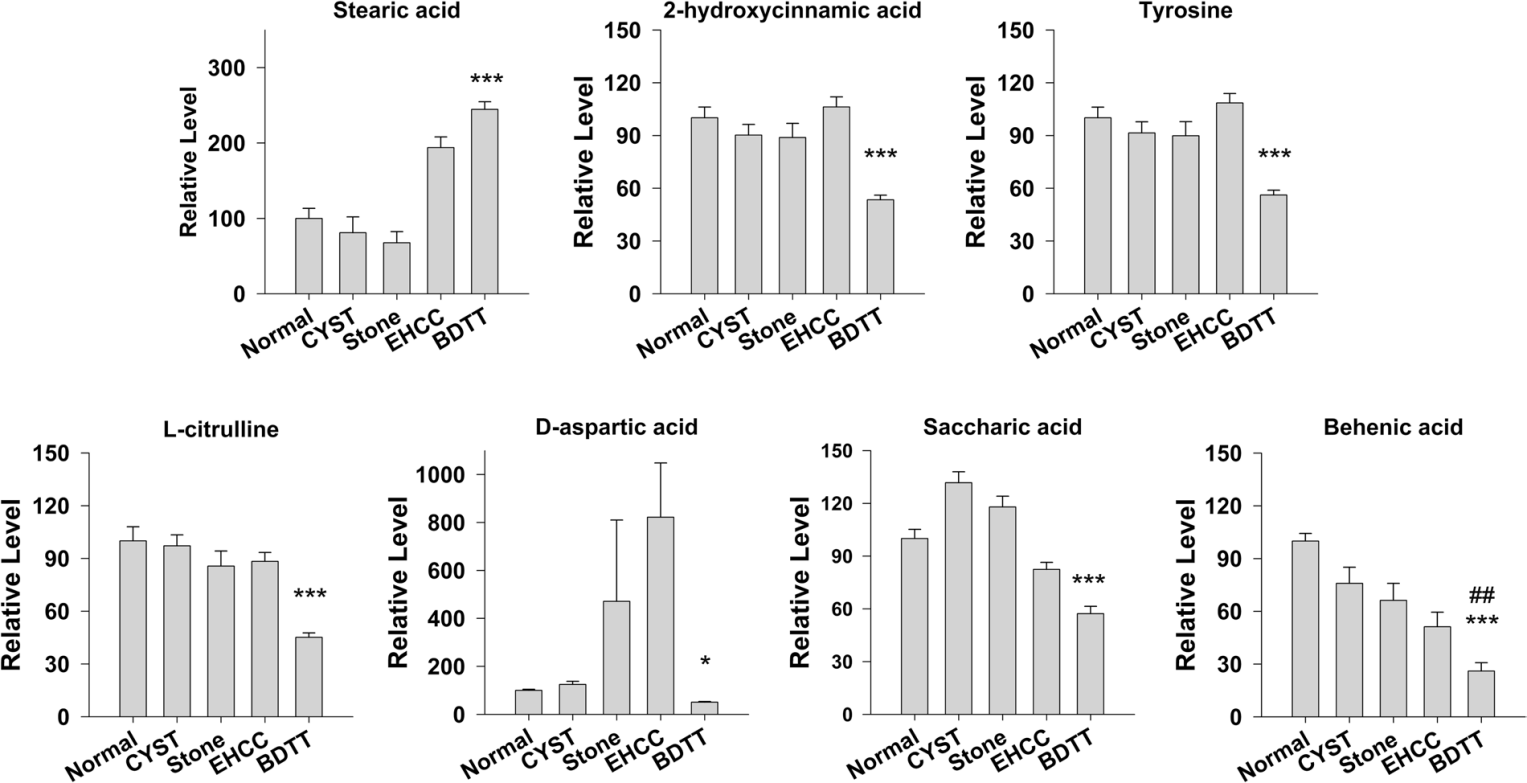
Several metabolites are significantly changed in BDTT group compared with other groups. Only Stearic acid is significantly increased in BDTT patient. Other 6 metabolites are significantly decreased. ^##^P < 0.01 compared with EHCC. *P < 0.05, ***P < 0.001 compared with other groups.

To confirm that the metabolites in Fig. 1B are really different between BDTT and other groups, we analyzed the patient serum samples in Table 2 by targeted measurement of the levels of these metabolites by UHPLC-QQQ-MS method. The PCA plot showed distinct distribution of those different samples (Fig. 2A). In order to obtain a higher level of group separation and get a better understanding of variables responsible for classification, OPLS-DA analysis was applied. Afterwards, the parameters for the classification from the software were high R2Y value and high Q2Y value, which were stable and good to fitness and prediction. Bi-plot analysis showed us that several metabolites were significantly changed in BDTT group compared with other groups, and these changes are also consistent with GC-MS results (Fig. 2B). All of the metabolites were normalized to normal group, and the level of those significantly changed metabolites in BDTT was plotted in Fig. 3. In those metabolites, only stearic acid is significantly increased in BDTT group compared with other groups. 2-hydroxycinnamic acid, tyrosine, L-citrulline, saccharic acid, D-aspartic acid and behanic acid, are significantly decreased in BDTT group.

### Several metabolites were identified as potential diagnosis biomarkers to distinguish BDTT from Stone, Cyst and EHCC

To further determine which metabolite can be used as a biomarker for BDTT diagnosis, we conducted ROC curve analysis. Though it is very easy to tell the difference between BDTT and normal people, we also firstly conducted ROC curve analysis between them. As it is shown in Table 3, all the metabolites showed very high sensitivity and specificity. Besides, since BDTT is often misdiagnosed as other bile duct disease, we then conducted ROC curve analysis between BDTT and several other bile duct diseases. Firstly, we analyzed which metabolite can be used as a biomarker to distinguish BDTT from Stone. The results showed that stearic acid, the only increased metabolite in BDTT patients as compared with other diseases, shows a very high sensitivity and specificity of 91.9% and 100%, respectively, suggesting that the level of stearic acid in serum can be used as reliable biomarker to distinguish BDTT from Stone. In addition, the decrease of L-citrulline, saccharic acid and D-aspartic acid can also be used as effective biomarkers for BDTT diagnosis, which provides an AUC (Area under the Curve of ROC) of 0.916, 0.973 and 0.952, respectively (Table 4). Furthermore, the specificity for L-cirtulline, saccharic acid and D-aspartic acid is 86.49%, 100% and 94.6%, respectively; and the same sensitivity of 88.89% for all three metabolites. However, the other three significantly changed metabolites, 2-hydroxycinnamic acid, tyrosine and behanic acid, are not good candidates because of their relatively low specificity (Table 4).

**Table 3:** ROC curve analysis of BDTT and Normal.

	AUC	Sensitivity	Specificity	Best cut-off	P-value
Stearic acid	0.978	83.8%	100%	29.46	4.20678E-06
2-hydroxycinnamic acid	0.969	100%	89.19%	749	6.48528E-06
Tyrosine	0.954	90%	89.19%	23.9	1.25992E-05
L-citrulline	0.959	90%	94.59%	6.025	9.91864E-06
Saccharic acid	0.930	90%	91.89%	292.5	3.58046E-05
D-aspartic acid	0.954	100%	89.19%	5.595	1.25992E-05
Behenic acid	0.966	100%	89.19%	67.31	7.32828E-06

**Table 4:** ROC curve analysis of BDTT and Stone.

	AUC	Sensitivity	Specificity	Best cut-off	P-value
Stearic acid	0.992	91.9%	100%	25.27	2.23565E-06
2-hydroxycinnamic acid	0.9249	100%	75.68%	664	5.01285E-05
Tyrosine	0.9219	100%	72.97%	20.5	6.25339E-05
L-citrulline	0.9159	88.89%	86.49%	5.09	4.48384E-05
Saccharic acid	0.973	88.89%	100%	374	7.78812E-06
D-aspartic acid	0.952	88.89%	94.59%	7.56	1.25992E-05
Behenic acid	0.7928	88.89%	78.38%	37.94	0.002796333

EHCC is another disease that is very likely to be confused with BDTT. Thus we performed ROC curve analysis to look for a good target to tell the differences between BDTT and EHCC. We found that at least five metabolites, including 2-hydroxycinnamic acid, tyrosine, L-citrulline, D-aspartic acid and behenic acid, can be used as good biomarkers. The sensitivity and specificity are shown in Table 5. However, stearic acid, which is predicted to be a very good marker to separate BDTT from Stone, only has a sensitivity of 81.8% and specificity of 61.3% (Table 5); therefore cannot clearly distinguish BDTT from EHCC. Then, we conducted ROC curve analysis between BDTT and Cyst. Except behenic acid, we found that the other metabolites all show very high sensitivity and specificity, and are very effective in distinguishing BDTT from Cyst (Table 6).

**Table 5:** ROC curve analysis of BDTT and EHCC.

	AUC	Sensitivity	Specificity	Best cut-off	P-value
Stearic acid	0.698	81.1%	61.3%	30.43	0.005187037
2-hydroxycinnamic acid	0.9259	83.87%	94.6%	873	1.79682E-09
Tyrosine	0.939	83.87%	94.59%	26.2	5.65034E-10
L-citrulline	0.9486	96.77%	83.78%	4.9	2.36822E-10
Saccharic acid	0.7803	58.06%	97.29%	303	7.53222E-05
D-aspartic acid	0.9895	96.77%	94.6%	6.73	4.70924E-12
Behenic acid	0.6857	100%	32.43%	6.418	0.008721231

**Table 6:** ROC curve analysis of BDTT and Cyst.

	AUC	Sensitivity	Specificity	Best cut-off	P-value
Stearic acid	0.954	100%	90%	17.19	1.25992E-05
2-hydroxycinnamic acid	0.9568	100%	89.19%	762	1.11825E-05
Tyrosine	0.95	100%	89.19%	24	1.50496E-05
L-citrulline	0.9757	100%	97.3%	6.93	4.76443E-06
Saccharic acid	0.9892	90%	100%	343	2.54027E-06
D-aspartic acid	0.973	100%	89.19%	6.19	5.39249E-06
Behenic acid	0.8649	90%	83.78%	46.88	0.000449528

In summary, our results showed that, there are several metabolites can be used to distinguish BDTT from each of other bile duct diseases that are likely to be confused with BDTT. However, not all of the metabolites showed high sensitivity and specificity in each condition. Interestingly, we noticed that L-citrulline and D-aspartic acid are not only can be used to distinguish BDTT from Stone, they are also effective in separating BDTT from two other diseases.

### Combination of L-citrulline and D-aspartic acid for BDTT diagnosis

**Figure 4:**
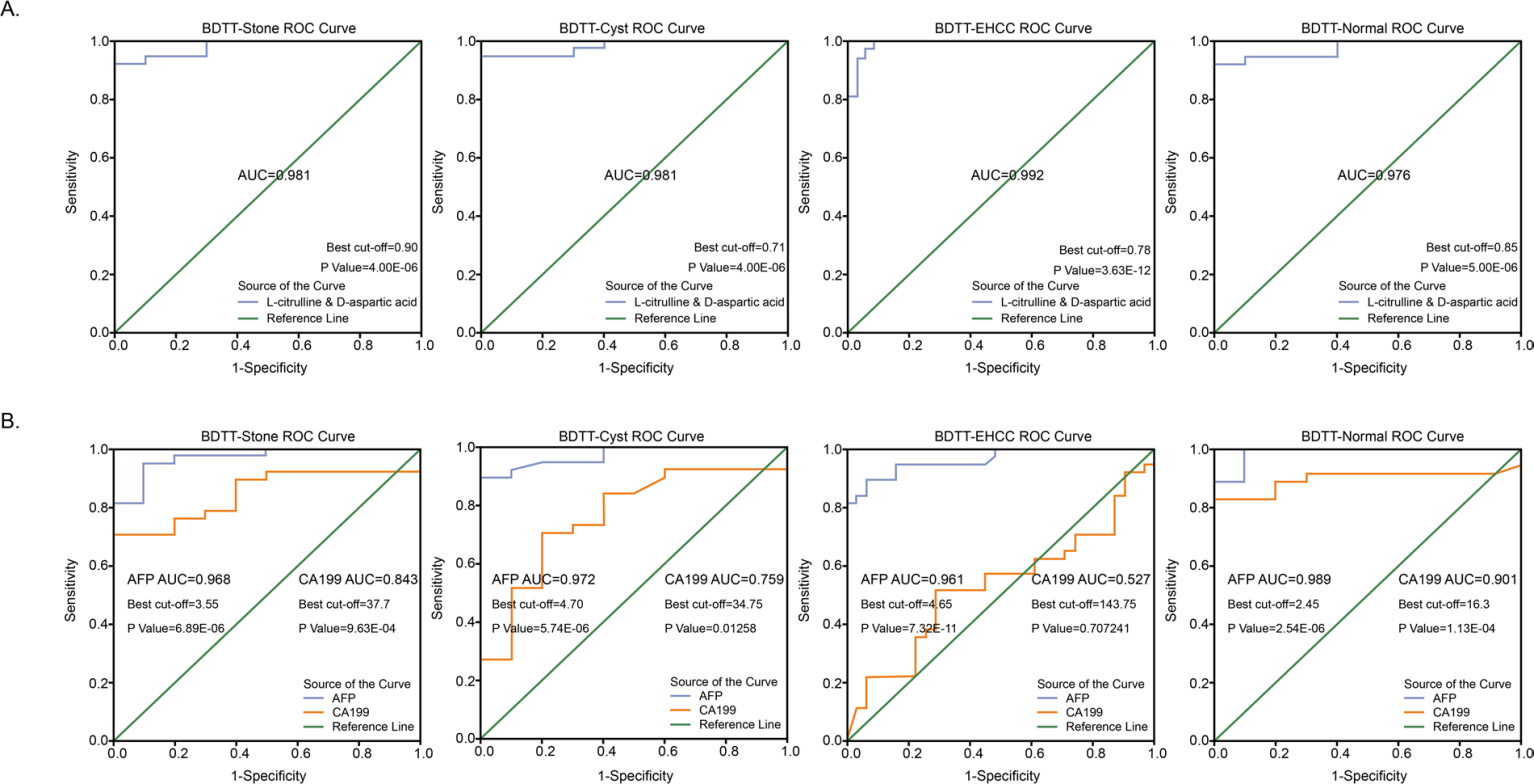
Combination of L-citrulline and D-aspartic acid for BDTT diagnosis has high sensitivity and specificity. (**A**) ROC curve analysis showed that the decrease of L-citrulline and D-aspartic acid concentration in serum could be good biomarker for distinguishing BDTT and other bile duct diseases. (**B**) ROC curve analysis showed the sensitivity and specificity of AFP and CA19-9, the widely used biomarkers for tumor diagnosis, for BDTT diagnosis.

Since the similar ROC curve analysis results were gotten for L-citrulline and D-aspartic acid individually for separateing BDTT from Stone, BDTT and EHCC, BDTT and Cyst, we performed another ROC curve analysis to examine whether the combination of the two metabolites can lead to better outcome. As it is shown in Fig. 4A, combination of these two metabolites gives a much better results than one metabolite alone. Both sensitivity and specificity have reached a level higher than 90% (The sensitivity for BDTT and Stone, BDTT and Cyst, and BDTT and EHCC is 91.9%, 94.6%, and 99.2%, respectively; and the specificity is 100%, 100% and 91.9%, respectively), and the AUC is also very close to 1.

AFP and CA19-9 are well-known and widely used serum biomarkers for tumor diagnosis. We analyzed the ROC curve of AFP and CA19-9 for BDTT diagnosis. As it is shown in Fig. 4B, when using the clinical cut-off value, the sensitivity for AFP in all of the three comparisons is as low as 54.1%, though the specificity is 100%. At the same time, the sensitivity and specificity for CA19-9 are also not very good (The sensitivity for BDTT and Stone, BDTT and Cyst, and BDTT and EHCC is 64.9%, 64.9%, and 70.3%, respectively; and the specificity is 80%, 100% and 25.8%, respectively). This result demonstrates that the combination of L-citrulline and D-aspartic acid is a potential tool for BDTT diagnosis, which may decrease the misdiagnosis of BDTT as other bile duct diseases.

### The level of L-citrulline is highly correlated with BDTT patient overall survival

Among the 37 BDTT patients, 35 patients accepted curative surgery, and the other 2 received palliative surgery. To be responsible for the patients, they were followed up until death or April 2015. We analyzed the survival data of these patients, and found that the serum level of L-citrulline and CA19-9 are strongly correlated with BDTT patient overall survival (Table 7). However, patient survival is not influenced by AFP, total bilirubin (TBIL), ALP and D-aspartic acid level. CA19-9, a classical marker for tumor diagnosis, is highly correlated with BDTT patient survival (Table 7). However, it is not a good marker for BDTT and EHCC diagnosis because it is highly upregulated in both BDTT and EHCC patients. Furthermore, it is worth to note that univariate analysis but not multivariate analysis shows a significant correlation of L-citrulline level and patient survival, suggesting L-citrulline is only suitable for diagnosis (Table 7). These results demonstrate that L-citrulline would be a good marker for BDTT diagnosis.

**Table 7:** Analysis of the variables related to Disease-free survival time (DFS) and Overall survival time (OS) in BDTT patients who underwent surgical resection with curative intent (n = 35)*.

Variables		n	Univariate analysis (Lon Rank Test)		Multivariate analysis (Cox’s regression model)			
			DFS	OS	DFS		OS	
			*P value*	*P value*	*P value*	*RR*(*95% Confidence interval*)	*P value*	*RR*(*95% Confidence interval*)
Gender	1: Male	30	0.192	0.986				
	2: Female	5						
Age (Years)	1: <60	30	**0**.**090**	0.660	**0**.**005**	**38**.**016** (**3**.**036 ~ 476**.**017**)		
	2: ≥60	5						
Tumor size (cm)	1: <5	15	0.583	0.837				
	2: ≥5	20						
Serum AFP (μg/L)	1: <20	16	0.503	0.233				
	2: 20~400	7						
	3: ≥400	12						
Serum CA19-9 (U/L)	1:<39	10	**<0**.**001**	**0**.**006**	**0**.**007**	**10**.**879** (**1**.**947 ~ 60**.**778**)	**0**.**031**	**2**.**882** (**1**.**098~7**.**556**)
	2: 39~1000	20						
	3: ≥1000	5						
Serum TBIL (μmol/L)	1: <34.2	20	0.441	0.282				
	2:34.2~171	10						
	3: ≥171	5						
Serum ALP (U/L)	1: <129	11	0.173	0.124				
	2: ≥129	24						
Serum L-citrulline (μg/ml)	1: <4.9	29	0.201	**0**.**044**			0.111	
	2: ≥4.9	6						
Serum D-aspartic acid (μg/ml)	1: <6.19	31	0.604	0.228				
	2: ≥6.19	4						

*α_in_ = 0.10, α_out_ = 0.15.

## Discussion

In this paper, we analyzed the small molecule metabolites in the serum of BDTT, EHCC, Stone and Cyst patients and normal people by GC-MS method, and then we applied UHPLC-QQQ-MS method to confirm those altered metabolites. We found that several metabolites are significantly changed in BDTT patient as compared to normal people and other patients. Further analysis demonstrates that the combined use of L-citrulline and D-aspartic acid is a potential biomarker for BDTT diagnosis, which clearly distinguishes BDTT from the bile duct diseases, Stone, Cyst and EHCC. In addition, we also found that the serum L-citrulline level is correlated with BDTT patient survival, suggesting its potential role for diagnosis. Due to the difficulty in collecting patient samples, the sample size in some group is relatively small. Nevertheless, we still easily identified the significant changes in some metabolites in BDTT patients. Importantly, the difference can be validated by UHPLC-QQQ-MS method using a different cohort of patients, suggesting that those metabolites (Fig. 3) are generally altered in these patients. In the future, it is critical to perform independent studies with larger sample sizes to further support our conclusion and identify the variations.

When BDTT occurs, jaundice is a main clinical manifestation. Those patients will also have very significantly increased serum total bilirubin. The bile duct lesion location and biliary ducts dilatation features also can be observed in BDTT patients^15^. Those pathological features can be used to distinguish HCC with BDTT from HCC without BDTT. However, jaundice also happens on bile duct stone and carcinoma patients, and very obvious bile duct obstruction and biliary ducts dilatation features are also observed in other bile duct patients. Besides, biliary ducts dilatation also occurs in choledochal cyst patients. Those pathological similarities are the major reasons of misdiagnosis of BDTT with other bile duct diseases. In the current study, we found that L-citrulline and D-aspartic acid are significantly decreased in BDTT patients compared with normal people and other bile duct diseases. However, there’s no difference when compare EHCC, Cyst, Stone with normal people (Fig. 3), demonstrating the specific metabolism of L-citrulline and D-aspartic acid in BDTT patient. L-citrulline is a key metabolic intermediate in urea cycle. It is also a byproduct of nitric oxide synthase (NOS) when it catalyzes the formation of nitric oxide which acts as a signaling molecule in regulating many biological processes, such as angiogenesis, vascular tone^16^. D-aspartic acid is an amino acid that is found to be decreased in BDTT patient. It is worth to note that D-aspartic acid is also an important metabolic intermediate in urea cycle, and it is produced from citrulline^17^. Since both L-citrulline and D-aspartic acid are key intermediates in urea cycle, our results suggest that the change in urea cycle is a key feature of BDTT. Previous reports by Chen *et al*. demonstrated that L-citrulline is reduced in HCC patients when compared with normal control^18^, which is very similar with our data that L-citrulline is decreased in BDTT patients. The same feature for HCC without BDTT and HCC with BDTT is that both of them show impaired liver functions. In contrast, EHCC, Cyst and Stone are the patients without any defect or with minimal impairment in their liver function (Tables 1 and 2). Considering that urea cycle is primarily taking place in liver, our analyses strongly suggest that the impairment of liver function in BDTT patient but not in other bile duct disease patient results in the decrease of urea cycle and the intermediate metabolites L-citrulline and D-aspartic acid, which lead a new diagnosis strategy. Furthermore, the combination of other types of biomarkers involved in urea cycle, such as mRNA and protein expression, may further add the accuracy of detecting BDTT, or simplify the detection procedure.

In addition to L-citrilline and D-aspartic acid, several other metabolites were also identified as potential biomarkers (Tables 4, 5 and 6). For example, ROC curve analysis also showed that saccharic acid is a good maker for distinguishing BDTT from Stone. Among those changed metabolites (Fig. 3), 2-hydroxycinnamic acid was reported to function as an antioxidant that can induce antioxidant response by increasing the levels of superoxide dismutase (SOD), catalase (CAT), glutathione-s-transferase (GST), glutathione reductase (GR), and glutathione (GSH) in the brain, liver, and kidney^19^. Tyrosine is used to synthesis protein. It is a non-essential amino acid that is synthesized from phenylalanine by the enzyme phenylalanine hydroxylase^20^. Phenylalanine hydroxylase is a monooxygenase, which also contribute to cell redox regulation^21^. The change of 2-hydroxycinnamic and tyrosine suggests that the redox states regulation signaling in BDTT patients may be a good target for treatment.

Biomarker for tumor diagnosis has very important clinical meanings. The well-known and most frequently used tumor markers for tumor diagnosis, such as CA19-9 and AFP, have their limitations^22^. The specificity of AFP is very good when it is used as a diagnosis marker (about 100%), its sensitivity is only 54.3%^23,24^. Our results also showed that CA19-9 is not so sensitive for BDTT and EHCC diagnosis due to its high level in both BDTT and EHCC patients, though the level of CA19-9 is correlated with BDTT patient survival (Table 7). Thus, a good biomarker that has high sensitivity and specificity is urgently needed for BDTT diagnosis. In this study, we analyzed small molecule metabolites in serum of BDTT, Stone, Cyst and EHCC patients, and calculated the sensitivity and specificity of the differentially expressed metabolites. By combination of ROC analysis and survival analysis, we conclude that that the combination of L-citrilline and D-aspartic acid as a biomarker for BDTT has very high sensitivity and specificity. Our results provide a base to develop a new method for BDTT diagnosis in clinic.

## Electronic supplementary material

Supplementary table 1

Supplementary table 2

Supplementary table 3
